# The teacher-student relationship in inclusive contexts: the role of mentalization and closeness

**DOI:** 10.3389/fpsyg.2025.1579785

**Published:** 2025-07-31

**Authors:** Gelsomina Cecere, Ilaria La Penna, Francesco Cerciello, Antonella Cavallaro, Michela Ponticorvo, Luigia Simona Sica, Alessandro Frolli

**Affiliations:** ^1^Developmental Neuroscience Research Lab, Department of International Humanities and Social Sciences, Rome University of International Studies, Rome, Italy; ^2^Department of Humanities, University of Naples Federico II, Naples, Italy

**Keywords:** special education teacher, mentalization, relational closeness, emotional regulation, special educational needs, gender differences, education, school

## Abstract

The teacher-student relationship is a central element in the educational context and significantly influences students’ academic performance and socio-emotional well-being, with particular attention to teachers working with students with Special Educational Needs (SEN). Scientific literature has recognized several factors that determine the quality of this relationship, such as teachers’ mentalization abilities, their emotion regulation skills, and relational closeness. This study analyzed gender differences in the mentalization abilities of special education teachers and identified the main predictors of relational closeness in educational relationships. The results revealed that female teachers show greater interest in the mental and emotional states of students and tend to be less prone to misinterpretations of them. Moreover, mentalization competencies, particularly interest in and certainty about students’ mental and emotional states, positively predict relational closeness, while difficulties in emotional regulation and a tendency to misinterpret students’ mental and emotional states reduce such closeness. Future research should further explore the relational dynamics between special education teachers and students with Special Educational Needs, utilizing complementary tools for a more comprehensive and accurate assessment.

## Introduction

The teacher-student relationship is a central element in the educational landscape, exerting a significant influence on both the school experience and the academic outcomes of students (Pianta, 2001). The quality of this relationship affects not only academic success but also the emotional well-being and social development of students. When teachers have confidence in their students’ potential, their positive reactions tend to foster improvements in academic performance, creating a virtuous cycle that strengthens educational expectations (Rosenthal and Jacobson, 2000). It is well-established that teachers’ sensitivity to the socio-emotional and academic needs of children constitutes a crucial aspect of the relational dynamics between educator and student. Previous studies have highlighted that high-quality in-service training programs can significantly enhance the professional competencies of early childhood educators and, at the same time, promote positive developmental outcomes in children (Egert et al., 2018; Werner et al., 2016).

Over the past decade, most studies on teacher-student relational dynamics have utilized the Student-Teacher Relationship Scale (STRS; Pianta, 2001), a tool that assesses three fundamental dimensions: Closeness, Conflict, and Dependency, which define the behavioral patterns characterizing the educator-student relationship (Birch and Ladd, 1997; Pianta, 1994). This study specifically focuses on the dimension of Closeness, understood as an emotionally warm relationship that the teacher is able to establish with the student, which fosters positive attitudes toward school, promotes open communication, and stimulates student engagement (Birch and Ladd, 1997). Students who develop a relationship characterized by closeness tend to rely on the teacher as a resource for addressing and resolving their difficulties. They are also more likely to share emotions and experiences, particularly during moments of conflict or discomfort (Pianta et al., 2003). A higher degree of closeness in the relationship can, therefore, enhance learning and academic performance, leading to a more positive attitude toward school (Birch and Ladd, 1997), reducing behavioral problems, and promoting the development of relational skills and social abilities (Buyse et al., 2008; Pianta and Stuhlman, 2004).

In the educational landscape, particular attention must be paid to special education. Special Educational Needs (SEN) refer to students who experience learning difficulties, physical or developmental disabilities, behavioral, emotional, or communication disorders, and learning gaps. The evolution from the role of the traditional teacher to that of the support teacher represents a concrete response to the diverse needs of students, highlighting the importance of inclusive education. While traditional teachers apply standardized methodologies suited to homogeneous groups, support teachers provide personalized assistance, fostering an inclusive and equitable learning environment for all students.

Scientific research has explored the role of support teachers and their relationship with students. Fung et al. (2015) analyzed the impact of support teachers on students with special educational needs in traditional schools. The results demonstrated that the presence of these teachers can significantly contribute to improving students’ academic performance and emotional well-being, promoting a more inclusive and personalized learning environment. Dignath et al. (2022) studied the reciprocal perceptions of teachers and students regarding their relationship, highlighting that a positive relationship between support teachers and students is associated with higher academic engagement and motivation in children with special educational needs. Similarly, Uttl et al. (2017) reviewed a wide range of studies on teacher-student relationships, concluding that high-quality relationships are essential for students’ academic and social success, particularly those with special educational needs. Support teachers, therefore, play a key role in promoting and facilitating these relationships.

Recent scientific evidence has explored the relational dynamics between teachers and students, investigating their connection to mentalization abilities (Schwarzer et al., 2021; Swan and Riley, 2012; Valle et al., 2022). Mentalization is a metacognitive skill that involves the capacity to perceive, interpret, and attribute meaning to emotions, beliefs, thoughts, desires, and other intentional mental states, both of oneself and others, that explain human behavior (Allen et al., 2008; Bateman and Fonagy, 2016; Dimaggio and Lysaker, 2015; Fonagy and Luyten, 2009). While it is well-documented that competence in reflecting on emotional and mental states and emotional regulation improves the quality of interactions and relationships in parent–child dynamics (Meins, 1997; Sharp and Fonagy, 2008; Zeegers et al., 2017), there remains a need to promote specific mentalization interventions among education professionals, particularly in the early stages of their careers (Colonnesi et al., 2017; Mata et al., 2022).

In the educational context, during interactions, teachers and students create a network of perceptions and constructions that include feelings, judgments, beliefs, and mutual expectations (Pianta et al., 2003), which significantly influences the behaviors of both teachers and students (Stuhlman and Pianta, 2002). To structure a positive, healthy, and warm relationship with students, it is, therefore, essential that teachers possess strong mentalization skills, which also impact the process of emotional regulation in teachers (Schwarzer et al., 2021), a competence that should be promoted in students. Emotions, in fact, play a crucial role in learning processes (Boekaerts and Pekrun, 2016; Clore and Huntsinger, 2009; Fuentes-Vilugrón et al., 2024; Zeidner, 2014). Specifically, positive emotions foster students’ psychological and physical well-being, leading to better academic outcomes. Conversely, negative emotions can hinder academic performance and promote student disengagement. In this perspective, teachers are tasked with facilitating this process, supporting students in managing their emotions. To fulfill this task effectively, it is crucial that teachers are first able to manage their own emotions adequately. The role of emotional regulation is particularly critical for support teachers, who must manage and support students with specific difficulties. In other words, when teachers perceive situations to be out of control, they struggle to manage their emotional and cognitive evaluations, leading to compromised adoption of effective teaching strategies and a negative impact on students’ well-being (Kunter et al., 2013; Sutton and Wheatley, 2003; Talmor et al., 2005). In light of these considerations, it is essential to develop interventions for support teachers aimed at improving their emotional regulation skills, with the goal of enhancing both professional well-being and the effectiveness of inclusive teaching practices.

Despite significant neurofunctional differences in social cognition between genders, attributable to structural factors, neural connectivity, and hormones that influence interpersonal processes and relational dynamics (Proverbio, 2023), a gap remains in the literature due to the limited availability of studies documenting significant discrepancies in mentalization abilities between men and women. Calero et al. (2013), in a study conducted with children and adolescents aged between 9 and 15 years, found that girls scored significantly higher than boys on all tasks requiring the attribution of mental states. Similarly, Hiller et al. (2014) observed that young adult women demonstrated superior abilities in attributing mental states compared to their male peers, and this ability was strongly associated with higher levels of prosociality. Consequently, it is argued that women are more capable of attributing mental states to others and generating socially more appropriate responses than men (Blair, 2005; Petrovic et al., 2023; Russell et al., 2007).

Other studies highlight that the understanding of others’ mental states and emotions promotes social adaptation in both genders, but in girls, it appears to have a more pronounced influence on prosocial behaviors compared to boys. However, current literature has yet to systematically investigate the role of gender in the relationship between understanding mental states and emotions and prosociality during middle childhood, representing a significant gap that needs to be addressed (Eisenberg et al., 2013; Rosnay et al., 2008; Slaughter et al., 2015).

### Aims and hypotheses of the present study

The present study aims to better understand the psychological and relational variables of special education teachers in inclusive contexts. Specifically, it sets out to investigate two main objectives: (i) to identify whether there are gender differences in the mentalization abilities of special education teachers; (ii) to explore which psychological factors are associated with the perceived closeness between teachers and students. To this end, the study examines the potential influence of teachers’ mentalization capacities and emotional regulation abilities on their perception of relational proximity, while also considering gender as a possible moderating factor. This research aims to provide a more nuanced understanding of the psychological and interpersonal variables that shape the relationships established by support teachers in inclusive educational settings.

## Methods

### Participants

For the present study, the sample was recruited from teachers enrolled in a specialization course in educational support at our university. During the initial phase of their training program, and in the context of regular instructional activities, participants were presented with the scientific rationale of the research project, along with its objectives and aims. On this occasion, voluntary participation in the study was proposed. Following this, participants were guided to designated classrooms equipped with computers, where they completed the research protocol. To be included in the study, participants had to declare: (a) work in school with children with special educational needs (internships, volunteer etc.); (b) absence of neurological, neuropsychological or neurodevelopmental disorders; (c) italian as mother tongue. A substantial body of scientific literature has demonstrated that individuals with neurodevelopmental disorders or specific neurological conditions may exhibit impairments in mentalization processes, executive functioning, and emotional regulation—central variables in the present study (Benzi et al., 2023; Gagliardini et al., 2023; Herold et al., 2015; Krämer et al., 2021; Luyten et al., 2020; Santoro et al., 2021). For this reason, the online survey included closed-ended questions such as: “Have you had psychiatric disorders?,” “Have you had neurological disorders?,” and “Have you had neurodevelopmental disorders?.” For each question, in addition to responding “yes/no,” participants were required to specify which ones. We initially recruited 467 participants, but 67 were excluded because they reported the presence of neurological, neuropsychological, or neurodevelopmental disorders. The final sample therefore consisted of 400 participants (77 males and 323 females, mean age = 40.50 years, SD = 9.30). The research was conducted after participants had signed informed consent and in accordance with the Ethical Standards of the Declaration of Helsinki and the approval (ID 25/2024) of the Ethics Committee (CERUS) of Department of International Humanities and Social Sciences, Rome University of International Studies (UNINT). Data were analyzed by the Developmental Neuroscience Research Lab at the Department of International Humanities and Social Sciences, Rome University of International Studies. The research protocol was digitized using the Microsoft Forms platform to facilitate efficient and user-friendly administration of the questionnaires. The form was completely anonymous to protect participants’ privacy. The research protocol included a sequence of three questionnaires, which were presented in the following order: the Teacher Reflective Functioning Questionnaire (TRFQ), the Difficulties in Emotion Regulation Scale (DERS), and the Closeness subscale. The entire protocol took approximately 40 min to complete. The data collected from these questionnaires were subsequently analyzed by the Developmental Neuroscience Research Lab, ensuring a rigorous approach to data management and analysis.

### Measures

The Student–Teacher Relationship Scale (STRS) is a self-assessment tool composed of 28 items, developed with reference to Attachment Theory, particularly the Attachment Q-set (Waters and Deane, 1985). Respondents rate each item on a five-point Likert scale, ranging from 1 (definitely does not apply) to 5 (definitely applies). The scale identifies three key factors: Conflict, Closeness, and Dependency. The Conflict dimension captures the negative aspects of the relationship, such as discordant interactions and the absence of a satisfactory bond between teacher and student. Specifically, high scores on this scale indicate that the student is perceived as being in opposition to the teacher. The Dependency dimension assesses the teacher’s perception of the student’s level of dependence on them. Specifically, high scores on this scale suggest that the student is perceived by the teacher as extremely dependent, with a tendency toward possessive or overly attached behaviors. Finally, the Closeness dimension explores the teacher’s perception of the level of affection and warmth in the relationship with the student. High scores on this scale indicate that the relationship fosters positive attitudes, encourages open communication, and promotes student involvement and engagement. Students characterized by Closeness are more likely to seek the teacher’s support in facing and resolving difficulties, as well as to share their emotions and experiences, particularly during challenging moments (Pianta et al., 2003). The original version of the instrument by Pianta (2001) was adapted and validated for use in the Italian context (Fraire et al., 2013). The Italian adaptation consists of 22 items, reinforcing the psychometric robustness of the STRS and confirming its three-dimensional structure: Closeness (α = 0.86), Conflict (α = 0.91), and Dependency (α = 0.69).

In the Italian context, Settanni et al. (2015) examined psychometric properties of a Short-Form of the questionnaire that confirmed just two dimensions (Closeness and Dependency). In this study, we considered just Closeness subscale (Cronbach’s alphas: 0.86; Settanni et al., 2015) composed of 6 items.

The Difficulties in Emotion Regulation Scale (DERS; Gratz and Roemer, 2004) is a self-assessment tool designed to evaluate clinically significant challenges in managing negative emotions. The Italian version of the questionnaire, developed by Sighinolfi et al. (2010), was utilized for this study. The instrument is composed of 33 multiple-choice items, which are grouped into six subscales: Lack of Acceptance, which reflects the tendency to experience secondary negative emotions or to react non-acceptingly to one’s emotional distress; Difficulty in distraction, which captures difficulties in maintaining concentration and completing tasks when faced with negative emotions; Lack of confidence, representing the perceived ability to effectively manage and regulate negative emotions; Lack of control, which identifies problems in maintaining behavioral control during emotional distress; Difficulty in recognition, reflecting the extent to which an individual can identify the emotion they are experiencing; and Reduced Self-Awareness, which includes items related to the ability to attend to and recognize one’s emotional states. The DERS provides both a total score, calculated by summing the 33 items (range: 33 to 132), and individual scores for each of the six subscales, allowing for an evaluation of specific aspects of emotional regulation difficulties (DERS_total). The internal consistency of the subscales, as measured by Cronbach’s alpha, is reported as 0.88, 0.85, 0.85, 0.83, 0.81, 0.74, and 0.90 for Lack of Acceptance, Difficulty in distraction, Lack of control, Reduced Self-Awareness, Lack of confidence, Difficulty in recognition, and the Total Score, respectively (Sighinolfi et al., 2010).

The Teacher Reflective Functioning Questionnaire (TRFQ) is a self-report instrument created to assess reflective functioning (RF) in teachers. This tool is based on the Parental Reflective Functioning Questionnaire (PRFQ; Luyten et al., 2017), originally developed by Luyten and colleagues to measure parents’ capacity to reflect on their children’s mental states. Similarly, the TRFQ evaluates teachers’ ability to understand and reflect on the mental states and emotional experiences of their students, a key skill for fostering a supportive and responsive learning environment. The TRFQ retains the same three subscales as the PRFQ, each assessing a specific but related aspect of reflective functioning: (a) Pre-mentalizing Mode (TRFQ-PM), it evaluates a teacher’s inclination to misinterpret or oversimplify students’ mental states. High scores suggest that the teacher might attribute students’ behaviors to manipulative or negative intentions rather than understanding their underlying causes; (b) Certainty about Mental States (TRFQ-CM), it measures the extent to which teachers feel certain about their understanding of students’ thoughts, emotions, and intentions. While a moderate level of certainty is beneficial, excessively high scores might indicate overconfidence, which could limit the teacher’s openness to alternative perspectives; (c) Interest and Curiosity in Mental States (TRFQ-IC), it reflects the teacher’s interest in understanding the thoughts, emotions, and mental states that drive student behavior. Higher scores indicate a more reflective and inquisitive approach, suggesting the teacher actively seeks to understand students’ inner experiences. The TRFQ is composed of 14 items that are rephrased to fit the educational context. Teachers are asked to indicate their level of agreement with each statement on a 7-point Likert scale, ranging from 1 (completely disagree) to 7 (completely agree). The subscale scores for TRFQ-PM, TRFQ-CM, and TRFQ-IC are calculated similarly to the PRFQ: mean of items included in the subscales.

### Statistical analysis

All the statistical analysis were performed using the Open Access software Jamovi version 2.6 (R Core Team, 2024; The Jamovi Project, 2024). We conducted a descriptive analysis to investigate how the variables are distributed. The variables are non-normal distributed.

Moreover, we conducted a non-parametric t-test for independent samples (Mann–Whitney U test) based on the gender of the sample to assess any gender differences in teachers’ mentalization skills as measured by the TRFQ.

Finally, we conducted a Generalized Linear Model considering as quantitative dependent variable the Closeness scale and as quantitative independent variables the subscales of the TRFQ and the ineffectiveness of emotion regulation as measured by the global score on the DERS.

## Results

Independent samples Mann–Whitney U test revealed significant gender differences in specific domains measured by the TRFQ questionnaire. In the “TRFQ-PM” domain, we found a significant difference between males and females (U = 8438.000, *p* < 0.001, rank biserial-correlation = 0.321) as well as in the “TRFQ-IC” domain (U = 8170.000, *p* < 0.001, rank-biserial correlation = −0.343). No significant gender differences were observed in the “CM” domain (U = 12069.500, *p* = 0.688, rank-biserial correlation = 0.029). Descriptive statistics for the TRFQ domains further highlight these gender-based differences. In the “PM” domain, females (n = 323) had a mean score of 2.001 (SD = 0.911), while males (n = 77) had a mean score of 2.595 (SD = 1.141). For the “IC” domain, females had a mean score of 5.911 (SD = 1.048) compared to 5.275 (SD = 1.161) for males. The “CM” domain showed minimal differences between females (mean = 3.296, SD = 1.209) and males (mean = 3.347, SD = 1.232). We reported descriptive statistics in Table 1.

**Table 1:** Means and standard deviations of TRFQ subscales based on gender.

TRFQ	Gender	*N*	Mean	SD	*p*
PM	Female	323	2.001	0.911	<0.001
	Male	77	2.595	1.141	
CM	Female	323	3.296	1.209	0.688
	Male	77	3.347	1.232	
IC	Female	323	5.911	1.048	<0.001
	Male	77	5.275	1.161	

PM, Pre-mentalizing Model; CM, Certainty about Mental States; IC, Interest and Curiosity in Mental States.

Finally, a Generalized Linear Model with a Gaussian distribution and identity link function was applied to analyze the factors predicting Closeness. The assumptions of the GLM model, including the distribution of residuals, have been verified and are reasonably fulfilled. The model included Gender, TRFQ subscales (PM, CM, and IC), and the DERS-TOT score as predictors. The analysis revealed that the model explained 24% of the variance in Closeness, as indicated by an R-squared value of 0.240. Additional fit indices supported the adequacy of the model, with an AIC of 764.279, a BIC of 808.185, and a deviance of 149.798. The residual deviance per degree of freedom (Chi-squared/DF) was 0.384, showing no evidence of overdispersion.

The loglikelihood ratio tests demonstrated that the TRFQ PM, CM, and IC subscales significantly contributed to predicting Closeness, with *p*-values below 0.001 for each variable. In contrast, the effect of Gender (*p* = 0.432) and DERS-TOT score (*p* = 0.245) were not statistically significant.

Parameter estimates further clarified these effects. The intercept of the model was significantly positive (3.645), representing the baseline level of Closeness when all predictors were at their mean values. The TRFQ PM subscale had a significant negative effect on Closeness (−0.175), with higher scores associated with reduced Closeness. Conversely, the TRFQ CM (0.090) and IC (0.165) subscales exhibited significant positive effects, suggesting that higher scores in these domains were related to greater Closeness. The effect of Gender, comparing males to females, was not significant (p = 0.432), indicating no substantial gender differences in Closeness. Finally, the DERS-TOT score had a no significant effect (Table 2).

**Table 2:** Generalized linear model of closeness scale as dependent variable.

Predictor	Estimate	CI 95%		Z	*p*
		Lower	Upper		
Intercept	3.645	3.558	3.732	82.172	<0.001
Gender	−0.070	−0.244	0.104	−0.786	0.432
TRFQ-PM	−0.175	−0.262	−0.088	−3.953	<0.001
TRFQ-CM	0.090	0.024	0.156	2.687	0.008
TRFQ-IC	0.165	0.095	0.236	4.589	<0.001
DERS-TOT	−0.003	−0.007	0.002	−1.161	0.246
Gender × TRFQ-PM	−0.057	−0.231	0.116	−0.648	0.517
Gender × TRFQ-CM	−0.078	−0.210	0.054	−1.158	0.248
Gender × TRFQ-IC	0.125	−0.016	0.266	1.739	0.083
Gender × DERS-TOT	0.010	0.001	0.020	2.164	0.031

PM, Pre-mentalizing Mode; CM, Certainty about Mental States; IC, Interest and Curiosity in Mental States; DERS-TOT, Difficulties in Emotion Regulation Scale.

**Figure 1:**
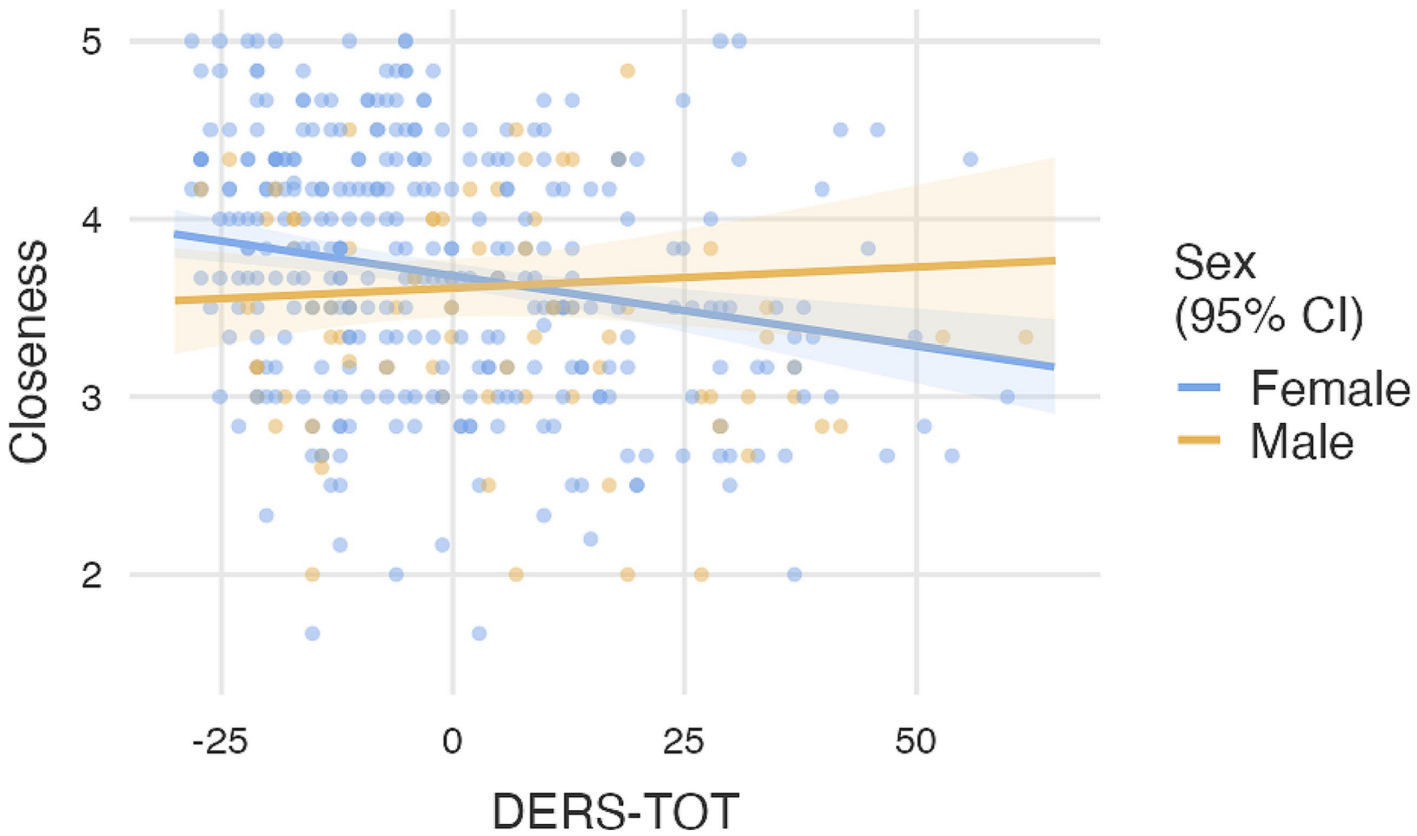
Scatter plots with the moderation effects of gender on relationship between DERS-TOT and closeness.

Moreover, Gender was included as a moderating variable by incorporating interaction terms between gender and each predictor. The only statistically significant interaction was between Gender and emotional dysregulation (DERS-TOT), β = 0.010, *p* = 0.031. This result suggests that the association between emotional dysregulation and perceived Closeness varies by Gender (Table 2). To clarify this moderation effect, we examined the simple slopes of DERS-TOT separately for female and male teachers. Among female teachers, emotional dysregulation was negatively associated with perceived closeness (β = −0.008, *p* < 0.001), indicating that higher levels of dysregulation corresponded to lower levels of perceived closeness. In contrast, among male teachers, this association was not significant (β = 0.002, *p* = 0.581), suggesting that perceived closeness was not meaningfully affected by emotional dysregulation in this group (Figure 1).

All other interactions did not reach statistical significance (*p* > 0.05), although the interaction between gender and TRFQ IC approached significance (*p* = 0.083), indicating a potentially meaningful trend.

## Discussion

The relationship between teacher and student constitutes a central element in the educational field, significantly influencing both academic performance and the emotional and social well-being of students (Pianta, 2001; Pianta and Nimetz, 2001). Over time, studies have examined interpersonal dynamics, highlighting the importance of mentalization skills (Pianta et al., 2003; Schwarzer et al., 2021; Stuhlman and Pianta, 2002; Swan and Riley, 2012; Valle et al., 2022) and the dimension of proximity, understood as the teacher’s ability to establish a warm relationship with the student, which fosters positive attitudes toward school, enhances learning, and strengthens relational competencies (Birch and Ladd, 1997; Buyse et al., 2008; Pianta et al., 2003; Pianta and Stuhlman, 2004). Therefore, this psychological construct represents a central competence in social cognition (Bateman and Fonagy, 2016; Desatnik et al., 2023; Fonagy and Luyten, 2009) and is associated with more effective emotional regulation (Ghanbari et al., 2023; Schwarzer et al., 2021). On the other hand, difficulties or psychopathologies (such as borderline personality disorder), where emotional dysregulation is a primary symptom, are treated through mentalization-based treatment (MBT; Bateman and Fonagy, 2010; Midgley et al., 2023). A crucial aspect of this context is special education, which addresses students with Special Educational Needs (SEN); support teachers play a fundamental role in improving the academic performance and emotional and social well-being of students with SEN (Dignath et al., 2022; Fung et al., 2015; Uttl et al., 2017). Emotional regulation competencies of teachers working in inclusive settings do not solely depend on their individual abilities but are also influenced by their perception of the controllability of the situation by the student and the student’s ability to cope with difficulties (Pekrun, 2006). In light of these scientific findings, the aim of the present study is to deepen the psychological and relational dynamics that characterize the role of support teachers within the educational context, in order to gain a more detailed and complex understanding of such interpersonal and cognitive processes.

The first objective of the present study was to examine gender differences in the mentalization ability of support teachers. The results reveal that females are less likely to misinterpret or oversimplify the mental states of students and show a greater interest in the mental and emotional states of students compared to males. Our findings are consistent with the few studies in the literature that document how females exhibit superior skills in all activities requiring the attribution of mental states to others, compared to males (Calero et al., 2013; Hiller et al., 2014). However, no gender differences emerged regarding the certainty of understanding the mental and emotional states of their students. This result could be interpreted in light of the growing professional awareness and sensitivity, regardless of gender, that characterizes teacher training (Spilt et al., 2018). Mentalization-related skills may result from uniform preparation focused on the relational and emotional aspects of teaching, which transcends gender differences. Furthermore, such uniformity may be influenced by a school environment that promotes inclusive and equitable pedagogical approaches, which require all teachers to develop skills in managing and interpreting the emotional and cognitive signals of students.

The second objective of this study was to identify the predictors of the proximity dimension in the teacher-student relationship. Using a generalized linear model, we found that the TRFQ subscales (PM, CM, IC) significantly contributed to predicting closeness, while the DERS total score alone did not emerge as a significant predictor. In this case, the effect of gender was not significant, suggesting that gender does not represent a determining factor in the perception of relational proximity. Statistical analyses revealed that the TRFQ-CM and TRFQ-IC subscales positively predict the proximity dimension. In other words, greater certainty and higher interest in understanding the mental and emotional states of students are associated with an increased perception of interpersonal proximity. Specifically, certainty regarding students’ mental and emotional states may lead the teacher to develop greater emotional security and a better understanding of interactions, which in turn fosters a sense of closeness in social relationships. On the contrary, the TRFQ-PM subscale negatively predicts the proximity dimension. Thus, the tendency to misinterpret or oversimplify students’ mental and emotional states led to less proximity between teachers and students. For example, it has been observed that teachers who tend to think that others have malevolent, hostile, or negative intentions tend to exhibit greater emotional distance in their interactions. However, results indicate that gender moderates the relationship between emotional dysregulation and teacher–student closeness. Specifically, emotional dysregulation plays a detrimental role for females, while it appears unrelated to closeness perceptions among males. This finding may reflect gender differences in emotional expression, relational needs, or teacher responsiveness. Dysregulated teachers tend to perceive relationships as distant and negative, as dysregulation can impair the ability to manage conflicts, communicate effectively, and respond empathetically to students’ emotions. Indeed, literature has highlighted the role of mentalization skills in the development of positive interpersonal relationships (Bateman and Fonagy, 2016; Fonagy and Allison, 2014; Frith and Frith, 2003; Peterson et al., 2005); thus, the connection between certainty and proximity can be explained by the fact that a correct and secure interpretation of students’ intentions and moods reduces uncertainties and conflicts, enhancing intimacy and cohesion between teacher and student. Moreover, the interest in understanding mental and emotional states is a key aspect of mentalization skills and social cognition: research suggests that when a person is interested in understanding others, they tend to develop deeper interpersonal relationships, as it fosters greater emotional openness and more sincere communication (Bateman and Fonagy, 2016; Desatnik et al., 2023; Fonagy and Allison, 2014; Frith and Frith, 2003). Malevolent mentalization, on the other hand, could lead to a range of defensive behaviors, such as mistrust, emotional avoidance, or aggressiveness, reducing the possibility of establishing a genuine connection.

### Limitations and future directions

The study presents several limitations. One limitation concerns the generalizability of the results, as the sample exhibited a significant gender imbalance, with a clear predominance of female participants; this limits the ability to draw conclusions about gender differences in a representative and broad manner. Another limitation is the use of self-report measures, which, although effective, may be susceptible to biases arising from the participants’ self-reflection. Moreover, unavailability of item-level data prevented us from calculating internal consistency indices (e.g., Cronbach’s alpha) for the measures used in this specific sample. Although the instruments employed have demonstrated good psychometric properties in previous studies, reliability can vary across populations and contexts. An additional limitation of the study concerns the inability to draw causal inferences between mentalization, emotion regulation, and relational closeness, due to the cross-sectional design employed, which does not allow for the analysis of directionality or dynamic processes over time. Furthermore, the impact of contextual variables such as the school environment, specific types of Special Educational Needs, student characteristics, and the duration of the teacher-student relationship was not considered; all of which could influence the perception of relational closeness and consequently affect the results.

It is also important to acknowledge that “students with SEN” is a highly heterogeneous group, encompassing a wide range of cognitive, emotional, and behavioral needs. Future research could explore whether these psychological and relational dynamics differ depending on the specific type or severity of student needs. However, the study also presents several strengths. First, the sample size and its careful selection: the participation of 400 teachers contributes to ensuring greater statistical robustness and superior external validity, allowing for broad generalization of the results. Additionally, the inclusion of support teachers without neurological or neuropsychological disorders further strengthens the reliability of the results. Second, the use of validated psychological tools designed to measure various aspects of mentalization, and emotional regulation allows for a thorough and rigorous analysis, providing solid grounding and validity to the collected data.

Finally, the study addresses a highly relevant issue within the educational context, with potentially significant implications for improving interpersonal relationships between support teachers and students with special educational needs, as well as for the revision and adjustment of teacher training programs. In particular, the focus on the mentalization of support teachers and the dimension of relational proximity represents an innovative and relevant approach within today’s educational landscape: the results obtained suggest that integrating mentalization strategies and enhancing relational proximity could be a tangible innovation in transforming the educational environment, making it more inclusive and responsive to the specific needs of students with Special Educational Needs. The awareness and ability of teachers to understand and respond empathetically to the emotional and cognitive experiences of students can foster a more positive relational climate, contributing to more effective and personalized teaching. In this perspective, our results offer insights for rethinking educational practices, highlighting the need to update teacher training programs so that educators can develop skills aimed at building meaningful relationships and adequately supporting students with difficulties.

In conclusion, future research should include a more balanced gender sample, thereby improving the generalizability of the results. Furthermore, it would be beneficial to integrate, alongside the tools used in this study, behavioral observations and ecological measures to ensure a more objective and comprehensive assessment of relational dynamics. Given the importance of the topic, further investigation is necessary to gain a more thorough understanding of the dynamics between support teachers and students with special educational needs within the school context.

## Data Availability

The raw data supporting the conclusions of this article will be made available by the authors, without undue reservation.
